# Association of *Schistosoma mansoni*-Specific IgG and IgE Antibody Production and Clinical Schistosomiasis Status in a Rural Area of Minas Gerais, Brazil

**DOI:** 10.1371/journal.pone.0088042

**Published:** 2014-02-04

**Authors:** Deborah Negrão-Corrêa, Juliana F. Fittipaldi, José Roberto Lambertucci, Mauro Martins Teixeira, Carlos Maurício de Figueiredo Antunes, Mariângela Carneiro

**Affiliations:** 1 Departamento de Parasitologia, Instituto de Ciências Biológicas, Universidade Federal de Minas Gerais, Programa de Pós-Graduação em Parasitologia, Belo Horizonte, MG, Brazil; 2 Departamento de Bioquímica e Imunologia, Instituto de Ciências Biológicas, Universidade Federal de Minas Gerais, Belo Horizonte, MG, Brazil; 3 Faculdade de Medicina Universidade Federal de Minas Gerais, Programa de Pós-Graduação em Ciências da Saúde: Infectologia e Medicina Tropical, Belo Horizonte, MG, Brazil; 4 Instituto de Ensino e Pesquisa, Santa Casa de Belo Horizonte, Belo Horizonte, MG, Brazil; Johns Hopkins University, United States of America

## Abstract

**Background:**

Studies in murine models and human populations have indicated that the collagen-rich granulomatous response against parasite eggs trapped in the liver is associated with the development of severe hepatosplenic schistosomiasis, characterized by periportal fibrosis and portal hypertension. The role of the humoral response in parasite susceptibility has been well established, but its participation in disease severity remains poorly understood. In this work, we evaluated the relationship between parasite-reactive IgE and IgG levels and schistosomiasis morbidity in infected patients with similar parasite burdens.

**Methodology/Principal Findings:**

Ninety-seven *Schistosoma mansoni*-infected individuals were subjected to clinical examination and abdominal ultrasound analysis. IgG reactivity and IgE concentration against *Schistosoma mansoni* soluble egg antigens (SEA) and adult worm antigen preparation (SWAP) were evaluated by ELISA assay. Multivariable linear regression models were used to evaluate the relationship between parasite-reactive antibodies and the co-variables investigated. The study population showed low parasite burden (median 30 eggs/g feces), constant re-infection, and signs of fibrosis was detected in more than 30% of individuals. Most infected individuals showed IgG reactivity, and the median concentrations of IgE anti-SEA and anti-SWAP antibodies were 1,870 and 1,375 ng/mL, respectively. There was no association between parasite burden and antibody response or any parameter of disease severity. However, IgG anti-SWAP level was positively associated with morbidity parameters, such as spleen size and thickness of portal vein at the entrance and secondary branch. In contrast, the data also revealed independent inverse correlations between concentration of parasite-reactive IgE and gallbladder wall thickness, a marker of fibrosis in schistosomiasis.

**Conclusions/Significance:**

The data indicate that IgG anti-SWAP is positively associated with severe schistosomiasis, independently of parasite burden, while high production of parasite-specific IgE is associated with mild disease in the human population. Antibody profiles are good correlates for schistosomiasis severity and could be tested as biomarkers of disease severity.

## Introduction

*Schistosoma mansoni* is the most prevalent species of the *Schistosoma* genus infecting human beings. Infection with this organism causes intestinal and hepatic schistosomiasis in more than 100 million individuals that primarily live in sub-Saharan Africa, the Caribbean and South American areas, including Brazil [1]–[3]. In endemic areas of *Schistosomiasis mansoni*, most infected individuals are asymptomatic or have mild clinical manifestations. However, in a minority of infected individuals, infection with this parasite can lead to severe hepatosplenic schistosomiasis, characterized by periportal fibrosis, portal hypertension, gastrointestinal bleeding and death [3]–[5].

Most of the morbidity related to chronic schistosomiasis is associated with hepatic and intestinal granulomatous inflammation induced by the parasite eggs that become trapped in these tissues. Granulomatous inflammation is dependent on CD4^+^ T cells, leading to tissue eosinophilia and the activation of alternatively activated macrophages and myofibroblasts, which can increase extracellular matrix production and collagen deposition; this inflammation may also cause extensive portal fibrosis and obstructive vessel lesions and increase portal pressure [6]–[10]. Several factors might influence both the development and level of morbidity in an exposed population, among them the degree and length of exposure, the intensity of the infection, concurrent pathologies, host and parasite genetics and nutritional status, which have all been associated with disease severity [5]. However, because granuloma formation is an immune-mediated process, factors that influence the induction and modulation of the immune response against parasite egg antigens could also be determinants in the progression of severe schistosomiasis.

In the murine model, *Schistosoma* egg deposition induces a type-2 immune response, which is characterized by the production of IL-4, IL-5 and IL-13 cytokines that, in addition to IL-10, has been associated with the down-modulation of the initial type-1 immune response and granuloma formation [10]–[13]. In experimental models, these type-2 cytokines, particularly IL-13, have been associated with fibrogenesis and therefore with severe pathology [9], [14]–[16]. In humans, the regulation of liver fibrosis during schistosomiasis may be even more complex, with multiple mediators regulating disease progression. Epidemiologic studies have indicated that *S. mansoni* infected patients presenting with severe fibrosis have elevated levels of the chemokine CCL3 [17], [18], tumor necrosis factor (TNF)-alpha, IL-5 and IL-13 [19]–[22], whereas patients with low levels of fibrosis present with high levels of IFN-gamma and IL-10 [19], [20]. Association of Th2-biased cytokine responses with persistent hepatic fibrosis and its persistence after treatment were also identified in *S. japonicum* infected patients from the Philippines [23].

In contrast to the amount of knowledge about the role of cytokines in granuloma formation and their association with disease severity, the participation of antibody responses against *Schistosoma* infection on the progression of clinical disease has been poorly investigated. The importance of B cell and antibody responses in the pathology associated with schistosomiasis has been suggested from experimental infections of *S. mansoni* in B cell-deficient mice [24], [25]. In human populations, immunoepidemiologic studies have indicated that increased levels of anti-schistosome IgE are closely correlated with resistance to re-infection and that high levels of anti-schistosome IgG4 are correlated with increased susceptibility to the parasite [26], [27]. In contrast, there are very few clinical studies showing the relationship between specific antibody production and schistosomiasis severity. These studies have demonstrated a positive association between anti-schistosome IgG responses, particularly IgG4, and severe schistosomiasis [28], [29]. To better understand the role of antibody response in the pathology of schistosomiasis, we first quantified IgE concentration and then evaluated the association of parasite (SEA and SWAP)-reactive IgG and IgE with the clinical form of the disease, which was defined based on clinical and ultrasound examination of *S. mansoni*-infected patients selected from the endemic area of Corrego do Choro, Padre Paraíso city, Minas Gerais.

## Materials and Methods

### Ethical Considerations

The present study was reviewed and approved by the Ethical Committee of Federal University of Minas Gerais, Brazil (number 274/05). At the time of data collection, all participants or their legal guardians were required to sign an informed consent form. Independently of participation in the study, patients with confirmed *S. mansoni* infection received specific treatment (a single dose of oxamniquine at 15 mg/kg for adults and 20 mg/kg for children, since the treatment recommended by Brazilian authorities at the time of the diagnosis), and other diagnosed diseases were treated or directed for specialized treatment.

To obtain *S. mansoni* antigens used in the experiments mice were experimentally infected as detailed in antigen preparation. All animal procedures were approved by the animal-care ethics committee of the Federal University of Minas Gerais (Protocol # 158/2008) and were performed under the guidelines from COBEA (Brazilian College of Animal Experimentation) and strictly followed the Brazilian law for “Procedures for the Scientific Use of Animals” (11.794/2008).

### Study Population

The subjects used in this study were selected among *S. mansoni*-infected residents of Jequitinhonha Valley, in the northeast of Minas Gerais state (Brazil), an area endemic for schistosomiasis. The initial study evaluated 741 inhabitants 5 years of age or older from rural communities of Jequitinhonha Valley and revealed a prevalence of schistosomiasis of 73% [30]. The study area has no reported cases of malaria and the individuals were serologically negative to *Leishmania* infection [30].

### Data Collection

The methodology employed for the data collection has previously been described in detail elsewhere [30]. In brief, each participant answered a structured questionnaire containing social information and clinical history associated with schistosomiasis. At the time that the questionnaire was given, a blood sample and feces were also collected. Parasitological confirmation of *S. mansoni* infection was determined based on egg counting of two thick stool smears using the Kato-Katz technique [31]. In addition to the parasitological examination, each individual was submitted to a clinical and abdominal ultrasound examination performed by independent experts. The clinical examination included a general physical evaluation and treatment history. Abdominal palpation was performed with patients in the dorsal decubitus position during a deep breath by two experienced physicians. The liver and spleen were considered palpable when the liver and spleen borders were felt below the costal margins by both examiners. Abdominal ultrasound examinations were performed using a conventional portable diagnostic ultrasound instrument (EUB 200 ultrasound unit, Hitachi) with an electronic linear 3.5-MHz transducer [30], [32]. During the ultrasound examination, the examiner measured the liver, spleen and thickness of the portal vein and gallbladder wall [33], [34]. The data were then entered into databases.

For the current study, we randomly selected one of the rural communities, Córrego do Choro in Padre Paraíso city, from the original study. In the community we evaluated all *S. mansoni*-infected patients that were 14 to 68 years old and had provided plasma samples that were kept at −70°C, totalizing 97 subjects. Plasma samples from 8 non-infected donors were also used for IgG reactivity controls. For the individuals selected for the current study, morbidity was evaluated by clinical aspects and by the quantitative method defined by Niamey’s protocol (proposed in 1996 and revised in 2000 [33], [34]), involving the ultrasound measurements of the diameter and thickness of the portal vein wall and their branches and the gallbladder. US-measurements were used as continuous values and categorized after height-adjusted by the average of the study population. For the categorization it was considered: longitudinal spleen size (<120 and ≥120 mm), longitudinal measurements of the left (<85 and ≥85 mm) and right (median <90 and ≥90 mm) lobes of the liver, the portal vein diameter (<11 and ≥11 mm) and wall thickness (≤5 and >5 mm), the thickness of portal vein secondary branches (≤4 and >4 mm) and gallbladder wall thickness (≤3 and >3).

### *Schistosoma mansoni* Antigens

To obtain adult worms and eggs for antigen preparation, Swiss mice were infected subcutaneously with 100 cercariae of *S. mansoni* (LE strain) that had been maintained in the Laboratory of Schistosomiasis (ICB/UFMG) by successive passages in *Biomphalaria glabrata* and hamsters (*Mesocricetus auratus*).

Adult *S. mansoni* worms (male and female) were recovered by perfusion of the circulatory system of 6-week-infected mice [35], washed and suspended in ice-cold phosphate-buffered saline (PBS). The recovered worms were snap-frozen in liquid nitrogen and ground into a paste as described by Dunne et al. [27]. After being thawed, the homogenate was resuspended in PBS containing a cocktail of protease inhibitors (Boehringer Mannheim, Indianapolis, IN, USA) and centrifuged at 10,000×g for 1 h at 4°C.

*Schistosoma mansoni* eggs were recovered from the livers of 6-week-infected mice, and the clean egg solution was ground in cold PBS to obtain the soluble egg antigen (SEA) [26], [27]. The homogenate was also centrifuged (10,000×g for 1 h at 4°C). The protein content of the supernatant from each preparation was estimated, and each antigen preparation (SEA and SWAP) was aliquoted and stored at −20°C.

### Sepharose Conjugation to SEA and SWAP Antigens

Sepharose columns (Cyanogen bromide-activated Sepharose 4B, Sigma, St. Louis, MO, USA) were conjugated with *S. mansoni* SEA or SWAP as described by March et al. [36]. The conjugated columns were used to adsorb parasite-reactive antibodies from the plasma of *S. mansoni*-infected patients. Briefly, the Sepharose was extensively washed with 1 mM HCl solution, followed by 3 washes with conjugation buffer (0.1 M sodium bicarbonate, 0.5 M sodium chloride, pH 8.3). Each antigen preparation (SEA and SWAP) was dialyzed overnight against conjugation buffer and added to the washed Sepharose at 5 mg antigen/mL Sepharose. The Sepharose/antigen mixture was incubated for 4 h at room temperature, followed by 18 h at 4°C under constant and slow agitation. After protein conjugation, the Sepharose was washed with conjugation buffer and blocked with 1 M ethanolamine (pH 8.0) for 2 h. Next, the antigen-conjugated Sepharose was submitted to 6 wash cycles alternately using an acidic buffer (0.1 M acetate containing 0.5 M sodium chloride, pH 3–4) and basic buffer (0.1 M Tris-HCl containing 0.5 M sodium chloride, pH 8–9) and then stored in a borate buffer (BBS, 0.1 M boric acid, 0.03 M sodium borate, 0.14 M sodium chloride, pH 8.3) containing 20% ethanol to prevent microbial growth. As a control for non-specific binding of plasma protein, we also prepared Sepharose columns with no conjugated antigens that were washed and blocked as above.

### Plasma Adsorption to Parasite-conjugated Columns

Two hundred µL each of SEA-Sepharose, SWAP-Sepharose or control-Sepharose were transferred to 1.5 mL Eppendorf tubes. Plasma samples from each patient were diluted 1∶100 in PBS buffer, and 400 µL of the diluted plasma was added to the Eppendorf tube containing SEA-Sepharose, another 400 µL of the diluted plasma was added to the SWAP-Sepharose tube and 400 µL of the diluted plasma was added to the control-Sepharose tube. Tubes containing Sepharose with plasma were vortexed for 1 min and kept at 4°C for 24 h with occasional agitation. Each tube was then centrifuged (5,000 g for 5 min at 4°C), and the supernatant was gently collected for quantification of total IgE and reactive IgG. At the end of the adsorption process, we obtained a sample of SEA-adsorbed plasma, a sample of SWAP-adsorbed plasma and a sample of control-adsorbed plasma from each patient.

### *Schistosoma mansoni*-reactive IgG

The presence of IgG reactive against *S. mansoni* antigens in the plasma of each patient was determined by enzyme-linked immunosorbent assay (ELISA). Briefly, 96-well plates (Nunc-Maxisorb Nagle Nunc International, Rochester, NY, USA) were coated with 100 µL/well of 0.1 M carbonate-bicarbonate buffer (pH 9.5) containing 10 µg of antigen/mL (SEA or SWAP) and incubated overnight at 4°C. The plates were blocked for 1 h with PBS buffer containing 1% bovine serum albumin (BSA, Sigma). After the blocking procedure and between each incubation step, the plates were washed 5 times with PBS containing 0.05% Tween (Sigma). One hundred microliters per well of diluted plasma from each patient was added to the plate and incubated for 1 h at room temperature. SWAP- and SEA-adsorbed plasma samples, as well as the control-adsorbed plasma samples, from each patient were tested. For the ELISA test, plasma samples collected after the adsorption procedure were diluted 1∶2 in PBS containing 1% BSA. To establish the reactivity threshold in plasma of infected patients, plasma samples were collected from 8 health volunteers with no report of previous infection for schistosomiasis, and the absorbance average plus 2 standard deviations from these samples were used as a reference. Bound IgG was detected by horseradish peroxidase-conjugated goat anti-human IgG (Sigma) diluted 1∶5,000 in PBS, followed by the addition of substrate solution (4 mM ο-Phenylenediamine (OPD, Sigma) containing hydrogen peroxide in 0.05 M phosphate-citrate buffer (pH 5.0). The reaction was allowed to proceed for 15 min at room temperature and stopped with 4 N H_2_SO_4_ (50 µl/well). The absorbance was measured at 492 nm using an automated ELISA reader (Molecular Devices, Sunnyvale, CA). Each plasma sample was tested in duplicate. To evaluate the reproducibility of this assay, 15% of the plasma samples were randomly selected to be retested; each duplicate received a different number from the original sample.

### Total IgE Quantification

To quantify the total IgE concentration in the plasma of the study population, a commercial kit (Bethyl, Texas, USA) with an established protocol from the manufacturer optimized for the study conditions was used. Briefly, 96-well plates (Nunc-Maxisorb) were sensitized with 1 µg/well of capture antibody for IgE (catalog number A80-108A, Bethyl) in carbonate-bicarbonate buffer (pH 9.6) for 1 h at 4°C and blocked for 45 min with 200 µL/well block solution (50 mM Tris-HCl buffer containing 0.14 M sodium chloride and 1% BSA). Between each step, the plates were washed 3 times with Tris/NaCl/Tween wash buffer (50 mM Tris-HCl buffer containing 0.14 M sodium chloride and 0.05% Tween 20). *S. mansoni*-adsorbed plasma (SEA and SWAP) and control-adsorbed plasma from each patient was diluted 1∶2 in diluent solution (50 mM Tris-HCl buffer containing 0.14 M sodium chloride, 0.05% Tween 20 and 0.1% BSA), and 100 µL/well was added to the plates. Known concentrations of purified human IgE (1,000-7.8 ng/mL) were added to each plate to obtain a standard curve. Plasma samples and standards were incubated for 1 h at room temperature. Immunoglobulin E bound to the plates was detected by the addition of peroxidase-conjugated anti-human IgE (stock 1 mg/mL, catalog number A80-108P, Bethyl) at 1∶40,000 dilution in a diluent solution, followed by the addition of substrate solution (4 mg OPD/3 µL H_2_O_2_ in 10 mL of citrate buffer, pH 5). After 30 min, the reaction was stopped with 100 µL of 2 N sulfuric acid solution, and absorbance was determined using a 492 nm filter in the ELISA reader (Molecular Devices, EMax). For each patient, the amount of total IgE in control-adsorbed plasma, as well as in SEA- and SWAP-adsorbed plasma, was quantified in duplicate. The difference between the amount of total IgE obtained in the control- and SEA-adsorbed plasma from the same patient was considered to be the amount of SEA-specific IgE present in the patient. Similarly, the difference between the control- and SWAP-adsorbed plasma represented the amount of SWAP-specific IgE [37].

### Statistical Analysis

Databases were generated using EPI-INFO version 6.04, and statistical analyses were performed using STATA version 11.0 software (Stata Corporation, 2010). Normality tests were determined with the Shapiro-Wilk test, and non-parametric variables were normalized. Categorical variables were compared using the χ^2^ test, means were compared using Student’s *t*-test or analysis of variance (ANOVA), and the Kruskal Wallis test was used to compare medians. Additionally, Spearman correlation analysis was performed for continuous variables. Linear regression analysis were used to evaluate the relationship between each immunoglobulin (IgE and IgG) reactive to egg (SEA) and adult worm (SWAP) antigens of *S. mansoni* and the co-variables collected (social, clinical, ultrasound and parasitological). IgE values were transformed into base 10 logarithms.

Initially, simple linear regression analysis was used to compare each immunoglobulin with all of the co-variables collected. Variables with p values <0.25 were selected to construct the multivariable linear regressions models and US-measurements were used only as continuous values for the final model. Moreover, variables with low frequency and that showed co-linearity were excluded from multivariable analysis. Full models for each immunoglobulin were constructed with all of the independent variables selected for univariate analysis, and modeling was carried out through a backward process. The only variables remaining in the model were those that were statistically significant with a confidence interval (CI) of 95% and p-value <0.05. Results are reported as linear regression parameter estimates, along with 95% CIs for the effect of independent variables for the immunoglobulin of interest. Homoscedasticity of the final models were evaluated.

## Results

Forty-seven of the 97 individuals were male (48.5%). The ages ranged from 14–68 years, and age was equally distributed throughout the groups; the mean age was 33.3 years (SD 5.8), and the median was 30 years (IQR 26). The evaluated individuals weighed on average 52.8 kg (SD 10) and had a mean height of 157 cm (SD 9.8). At the time of sample collection, the population had no access to treated water or sewage treatment, and 32 individuals (33%) reported previous *Schistosoma* treatment. Seventy-seven individuals (79%) from the study population showed less than 100 *Schistosoma* eggs eliminated per g of feces (low parasite burden), with a mean of 81.1±163.4 and median of 30 eggs/g feces. Among the 97 individuals evaluated in the study, there was no association between parasite burden and age, sex or gender (Table 1).

**Table 1:** Characteristics of the study population, Corrego do Choro, Padre Paraíso, Minas Gerais (n = 97).

Characteristics	Number (%)	Parasite burdenEggs/g fecesMeans ± SD	p-value
Sex			
Male	47 (48.5)	89±148	0.959
Female	50 (51.5)	90±189	
Age group (years)			
14–19	27 (27.8)	137±283	0.482
20–29	18 (18.6)	61±107	
30–39	21 (21.7)	51±73	
40–49	14 (14.4)	96±120	
50–68	17 (17.5)	82±83	
*S. mansoni* treatment			
Yes	32 (33.0)	88.5±161	0.967
No	65 (67.0)	89.9±171	

The clinical examination revealed that among the 97 individuals evaluated, 7 (7.2%) reported previous hematemesis, and 2 had collateral veins detected during clinical examination. The spleen of 9 patients was enlarged, and 4 of them also showed hard consistency. Moreover, 50 individuals (51.6%) showed enlarged livers, with 10 of them having a hard consistency and 4 individuals showing gross irregularities (pseudo-nodules) of the liver surface.

The measurements of the spleen and left and right liver lobes sizes and the diameters and thickness of the portal vein and gallbladder wall thickness obtained by ultrasound evaluation are presented in Table 2. After categorizing the ultrasound measurements to evaluate *Schistosoma*-related morbidity, only 5 individuals showed spleen sizes larger than 120 mm, and 4 had right liver lobes that were reduced in size. However, at least 30% of the study population showed some signal of fibrosis, such as thickening of the portal vein and gallbladder wall (Table 2). Notably, there was no association between parasite burden and the parameters of parasite morbidity that were evaluated (Table 2).

**Table 2:** Parasite burden and ultrasound measurements of spleen, liver, portal vein and gallbladder of individuals evaluated from Corrego do Choro, Padre Paraíso, Minas Gerais (n = 97).

Organ evaluated – size in mm	Size (mm)Mean ± SD	N (%)	Parasite burden Eggs/gfeces Means ± SD	p-value
Spleen – Longitudinal size	84.6±26.7			0.280
<119		92 (94.8)	85.1±168.0	
≥120		5 (5.2)	169.5±197.0	
Liver – Longitudinal left lobes	97.0±18.5			0.458
<84		27 (27.8)	68.5±81.6	
≥85		70 (72.2)	97.6±192.9	
Liver - Longitudinal right lobes	119.6±17.8			0.957
<90		4 (4.17)	82.5±41.1	
≥90		92 (95.83)	87.1±171.9	
Thickness of the portal vein wall	5.3±2.0			0.481
≤5		57 (58.7)	106.6±207	
>5		40 (41.3)	65.2±89.1	
Diameter of portal vein	9.6±2.2			0.238
<11		65 (67.0)	74.2±168.9	
≥11		32 (33.0)	120.5±169.2	
Thickness of wall of portal vein Secondary branches	4.14±1.17			0.488
≤4		65 (68.4)	83.0±191.0	
>4		30 (31.6)	109.3±116.0	
Gallbladder - Thickness of wall	3.5±1.1			0.259
≤3		57 (59.4)	106.6±207.4	
>3		39 (40.6)	66.5±89.6	

**Figure 1:**
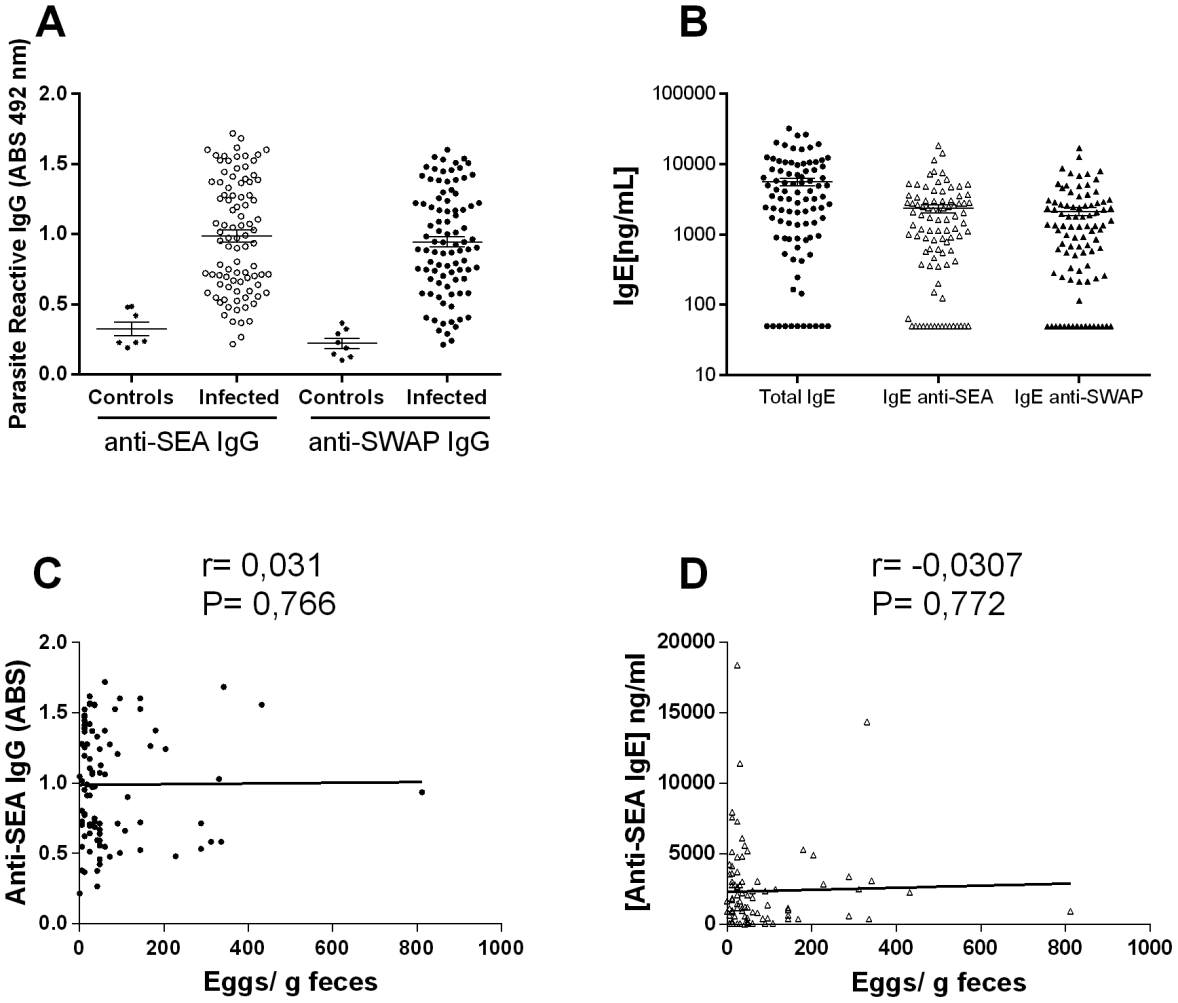
Parasite-reactive antibody in plasma of the evaluated individuals and its association with parasite burden. (A) Level of IgG anti-SEA and anti-SWAP antigens estimated by ELISA assay in plasma samples from *Schistosoma*-infected and uninfected controls. (B) Concentration of total IgE and parasite-reactive, SEA and SWAP, IgE in plasma samples of *Schistosoma*-infected individuals indirectly estimated by ELISA assay in samples before and after been submitted to antigen-specific adsorption. Correlation analyses between parasite burden, estimated by the number of eggs of *S. mansoni* eliminated in the host feces and production of IgG anti-SEA antigens (C) and IgE anti-SEA antigens (D). Spearman correlation coefficients and p-values, are shown for each graph.

Of the total 97 patients that were clinically evaluated, 91 individuals had plasma samples that were tested for IgG reactivity, and only 80 individuals had sufficient plasma volumes to perform the adsorption and measurement of IgE. Among these individuals, the average level of anti-SEA IgG was 0.988±0.401, and the level of anti-SWAP IgG was 0.945±0.370. Based on the cut-off established from the non-infected individuals, 73 *S. mansoni*-infected individuals showed elevated levels of anti-SEA IgG, and 80 had elevated anti-SWAP IgG (Fig. 1A). The adsorption of each plasma sample against SEA-conjugated Sepharose abolished the low IgE readings and resulted in significant reductions in IgG reactivity, from 0.988 to 0.463, the reactivity level of non-infected individuals. Similarly, plasma samples adsorbed against SWAP-conjugated Sepharose also showed IgG reactivity at the level of non-infected individuals (0.443), indicating that most of the parasite-reactive antibodies were removed from plasma. The adsorption of parasite-reactive antibodies allowed us to estimate the concentration of total IgE and SEA-reactive IgE or SWAP-reactive IgE in the plasma of *S. mansoni*-infected individuals. The median total IgE in this population was 4,140 ng/mL, and the median concentration of IgE anti-SEA and anti-SWAP was 1,870 and 1,375 ng/mL, respectively (Fig. 1B). In the population evaluated in this study there was no association between parasite-reactive IgG or IgE anti-SEA and parasite burden (Fig. 1C and 1D). Similar results were also observed with antibodies against SWAP antigens (data not showed).

**Figure 2:**
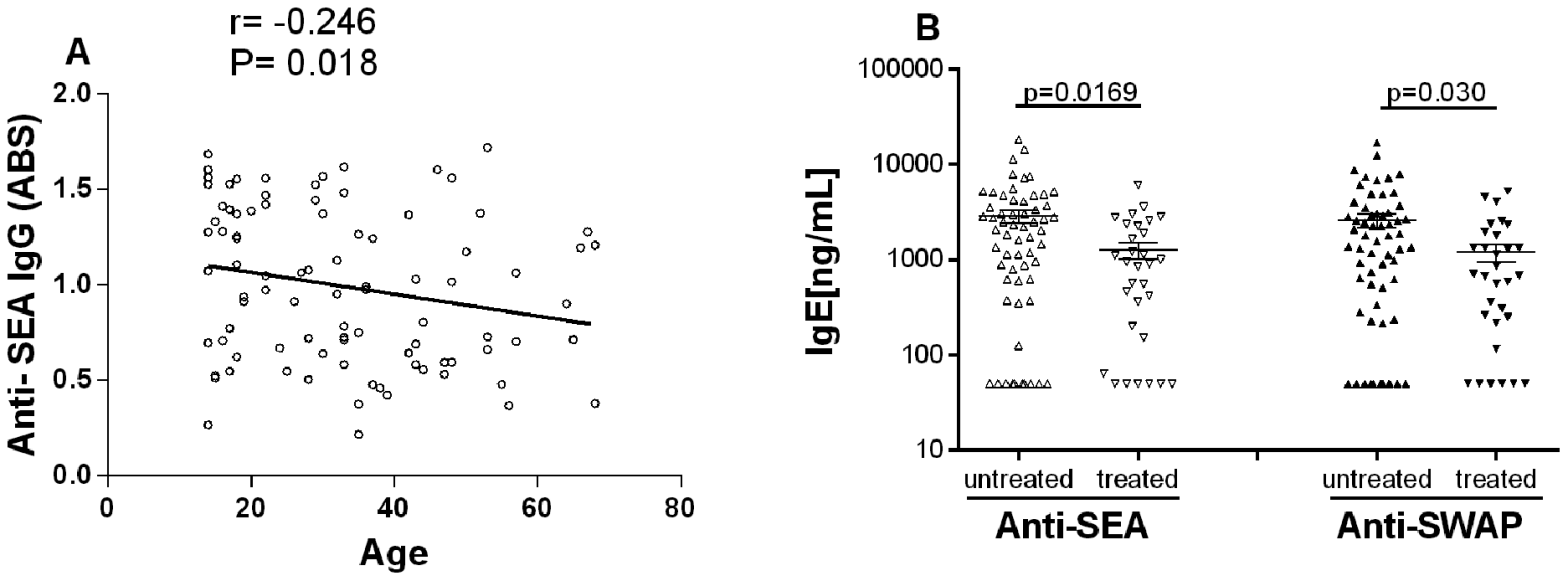
Association between parasite-reactive antibody production and host age (A) and schistosomiasis treatment (B). Demographic and social information were obtained from the questionnaire, and antibody levels were estimated by ELISA. There was an inverse correlation between host age and IgG anti-SEA levels, as shown by Spearman analysis (A). The concentration of parasite-specific IgE was significantly lower (Mann-Whitney test) in individuals that had received previous Schistosoma treatment and were re-infected (B). Antibody levels showed no association with other demographic and social factors evaluated.

Production of parasite-reactive antibodies was also associated with different demographic and social aspects and a negative correlation between IgG anti-SEA levels and age (r = −0.2248; p = 0.032) was observed (Fig. 2A). Individuals who reported frequent contact with natural sources of water (n = 75) had total IgE concentrations and levels of IgG anti-SEA that were significantly higher than the others. There was no association between IgG response (SEA or SWAP) and schistosome treatment. In contrast, infected individuals who had received treatment against *Schistosoma* infection (n = 30) showed a significant decrease in IgE response (Fig. 2B).

To investigate whether the antibody response was related to schistosomiasis morbidity, the concentration of parasite-reactive IgE or the level of IgG reactivity was associated with clinical and ultrasound parameters of disease. The level of IgG anti-SWAP was significantly higher in patients with livers of hard consistencies and gross irregularities on the liver surface. Moreover, increases in the size of the left lobe of the liver, as defined by clinical examination, were positively correlated with the level of IgG anti-SEA but not with IgG anti-SWAP, while individuals that had palpable spleens during clinical examinations showed higher levels of IgG anti-SWAP (data not shown). In contrast, there was no association between the concentration of IgE reactive against SEA or SWAP with any of the clinical parameters evaluated.

**Figure 3:**
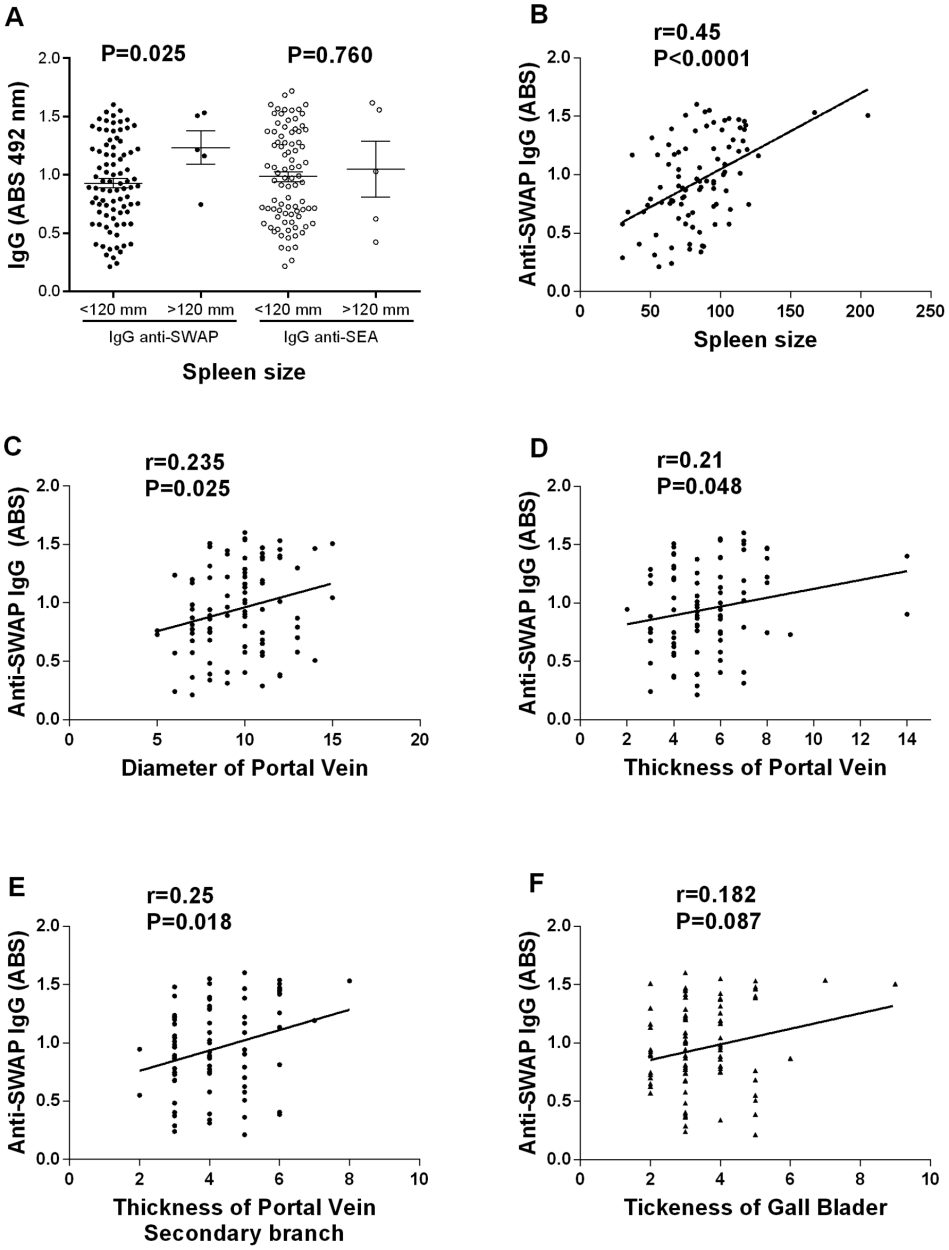
Association between level of anti-SWAP IgG and schistosomiasis-related morbidity. (A) Parasite-reactive IgG levels were associated with longitudinal spleen size, categorized as <120 or ≥120 mm, as analyzed by Mann-Whitney test. Correlation analyses were performed on the absorbance of anti-SWAP IgG levels by ELISA and the following morbidity parameters: longitudinal spleen size (B), diameter of the portal vein (C), thickness of the portal vein at its entrance into the *porta hepatis* (D) and its bifurcation inside the liver (E), and the thickness of gallbladder wall (F) determined by ultrasound evaluation. Spearman correlation coefficients and p-values are shown for each graph.

Liver size as determined by ultrasound examination showed no association with the level of IgG anti-SWAP or anti-SEA, but there was a positive association between the level of IgG anti-SWAP and the size of the spleen (Fig. 3A and 3B). There was no association between IgE concentration and liver or spleen size.

**Figure 4:**
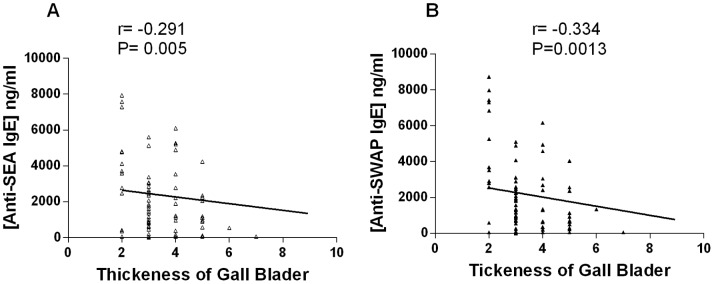
Association between parasite-reactive IgE and schistosomiasis-related morbidity. Correlation analyses were performed on the concentration of anti-SEA IgE (A) or anti-SWAP IgE (B) and the thickness of the gallbladder determined by ultrasound evaluation. Spearman correlation coefficients and p-values are shown for each graph.

Fibrosis induced by *S. mansoni* infection was evaluated by ultrasound by measuring the portal vein diameter and thickness at its entrance into the *porta hepatis* and its bifurcation inside the liver, as well as the thickness gallbladder wall. The association between these disease parameters and antibody production was also estimated. A positive association was observed between IgG anti-SWAP and the diameter and thickness of the portal vein at the entrance and its bifurcation (Fig. 3C, 3D, and 3E), but there was no significant association IgG and thickness gallbladder (Fig. 3F). In contrast, the concentrations of IgE anti-SEA and anti-SWAP were negatively correlated with gallbladder thickness (Fig. 4).

### Multivariable Linear Regression Models

Tables 3 and 4 show the final adjusted linear regression models that describe the effect of clinical, demographic and ultrasound parameters of parasite-specific IgG and IgE production. The analysis showed that parasite reactive (SEA and SWAP)-IgE concentration was inversely associated with thickness of gallbladder wall and with *Schistosoma* treatment (Table 3). Age was negatively associated with the level of IgG anti-SEA, while the thickness of portal vein wall at its entrance and its bifurcation inside the liver and spleen size measured by ultrasound were positively associated with the level of IgG anti-SWAP (Table 4).

**Table 3:** Anti-SEA and anti-SWAP IgE models for schistosomiasis patients, Minas Gerais.

	Anti-SEA IgE				Anti-SWAP IgE			
Variables	β	SE	95% CI	P	β	SE	95% CI	p
*Schistosoma*-Treated	−0.389	0.156	−0.699– −0.079	0.014	−0.359	0.149	−0.655– −0.060	0.019
Thickness of gallbladder wall	−0.200	0.070	−0.338– −0.059	0.006	−0.219	0.067	−0.354– − 0.085	0.002

β, coefficient estimates; SE, standard error.

**Table 4:** Anti-SEA and anti-SWAP IgG models for schistosomiasis patients, Minas Gerais.

	Anti-SEA IgG				Anti-SWAP IgG			
Variables	β	SE	95% CI	p	β	SE	95% CI	p
Age	−0.006	0.003	−0.010 – 0.001	0.028				
Thickness of portal vein wall					0.059	0.022	0.014–0.105	0.010
Thickness of wall atsecondary branch					0.027	0.013	0.001–0.052	0.040
Spleen size (Ultrasound)					0.004	0.001	0.002–0.0.001	0.000

β, coefficient estimates; SE, standard error.

## Discussion

This cross-sectional study evaluated the possible association between antibody production, IgG and IgE, and schistosomiasis in a naturally infected population. The major findings of the present study can be summarized as follows: i) A human population exposed to low *S. mansoni* burden and frequent re-infection can develop high level of morbidity, especially fibrosis. ii) Among the infected individuals, there was no association between parasite burden and antibody response or disease severity. iii) There was a positive association between parasite-reactive IgG (mainly anti-SWAP) level and both spleen size and portal vein thickness. iv) The data also revealed inverse correlations between the concentration of parasite-reactive IgE and gallbladder wall thickness, an important marker of fibrosis in schistosomiasis.

In the present work, we quantified for the first time in a human population the concentration of IgE against schistosome antigens without interference from other isotypes, such as IgG4. The procedure is essential to evaluate the real role of IgE in the infection, since B cells can switch sequentially from an IgG4-producing B cell into an IgE-producing B cell. Therefore, high IgG4 production, which may be more than 100-fold higher than IgE antibodies, can interfere with the detection of parasite-specific IgE [38]. In the serum of Schistosoma-infected patients, Rihet et al. [39] demonstrated that more than 90% of parasite-specific IgE is inhibited by IgG4, when measurements are performed by specific IgE ELISA. Therefore, the measurement of parasite-reactive IgE directly by an ELISA assay would underestimate the true amount of the antibody and compromise the evaluation of the role of IgE in protection or disease induction. In contrast to IgE, IgG is the predominant immunoglobulin class in plasma, and there is no experimental evidence of inhibition of parasite-reactive IgG antibody by any other immunoglobulin class [39], indicating that the use of a direct ELISA to estimate IgG reactivity levels against parasite antigens is appropriate.

The individuals evaluated in this study had a homogeneous gender and age group distribution, and no association between gender, age group and parasite burden was observed. Although most population analyses performed in high endemic areas for *Schistosoma* infection have shown an inverse correlation between host age and parasite burden [40], [41], [42], the individuals analyzed in the current study showed no such association. However, it is important to mention that we selected infected individuals ranging from 14 to 68 years old to avoid including the highly susceptible younger population and the highly resistant older ones. Another important characteristic of the study population was the low parasite burden, which was characterized by a median of 30 eggs per g of feces as quantified by the Kato-Katz technique. Likely, due to the low parasite burden detected in the individuals evaluated in the current study, there was also no association between parasite burden and disease severity. Most previous studies have shown that parasite burden is one of the most important factors in the development of severe schistosomiasis [4], [5], [40], [43], [44]; however, this association was reported in areas of high intensity of parasite infection. In *S. mansoni* hyperendemic areas, Bina and Prata [4] demonstrated a strong association between parasite burden and severe pathology in patients eliminating over than 1,000 eggs/g feces. In contrast, other authors [5], [45] have also reported severe forms of schistosomiasis in patients with low parasite burdens, demonstrating that parasite burden is not the only determining factor for disease progression. Endemic areas of *S. mansoni* with low parasite burdens and no association between parasite burden and disease severity, such as the area evaluated in the present study, offer a unique opportunity to evaluate the effect of the immune response on disease severity independently of infection intensity. More importantly, even with low parasite burdens, more than 30% of the individuals evaluated in this study had some measureable level of fibrosis, as shown in Table 2.

Regardless of the antibody response to this disease, most of the studies on *Schistosoma*-infected populations have reported that anti-*Schistosoma* IgG4 levels in infected children are associated with higher parasite burdens and parasite susceptibility [26], [43], [46], [47]. In the current study, there was no association between parasite-reactive IgG and age group or parasite burden of the infected individuals, and this finding could be a consequence of the low parasite burden in the study population. However, a negative correlation between anti-SEA IgG and age was identified, and this association was confirmed in the multivariable linear regression model.

In contrast to IgG, data from endemic areas of schistosomiasis have shown that the reactivity of anti-SWAP IgE increases with age [47], [48], but there have been no associations between IgE reactivity and the intensity of infection with *S. mansoni* [48]. Similarly, we also did not find an association between IgE production and parasite burden. Although the association between schistosome-specific IgE and parasite burden is not always found in endemic areas, high levels of schistosome-specific IgE have been frequently associated with protection against re-infection [26], [27], [49]–[52]. In highly endemic areas, the positive association of schistosome-specific IgE and CD23^+^ B cells with resistance to *S. mansoni* can be detected even in children [53]. Notably, we evaluated only infected individuals with low parasite burdens, and individuals who reported previous *Schistosoma* treatment had significantly lower levels of IgE, independently of age, sex or parasite burden. These data are suggestive of a protective role for IgE in the study population.

Recent data from a large casuistic study in an *S. mansoni*-endemic area of Bahia, Brazil, also showed no significant difference in the levels of *Schistosoma*-specific IgE between individuals with different parasite burdens, but the authors found that levels of anti-adult worm IgG4 and IgE/IgG4 ratios were inversely associated with *S. mansoni* parasite burden [54]. A significant negative association between the ratio of IgE/IgG4 and infection intensity was also detected in younger (5–18 years old) Zimbabweans in an *S. haematobium*-endemic area [55]. These data indicated that resistance against *Schistosoma* infection could be related to the IgE/IgG4 balance rather than the level of production of a single isotype.

Although there have been many epidemiological studies correlating antibody production with host susceptibility/resistance during *Schistosoma* infection, there have been very few studies showing the relationship between specific antibody production and parameters of disease severity. Using ultrasound measurements to categorize patients infected with *S. mansoni*, Tawfeek et al. [56] reported a significantly higher serum level of anti-SEA IgG4 in patients with periportal fibrosis and portal hypertension. A cross-sectional survey conducted in individuals of *S. mansoni*-endemic areas also showed that levels of anti-SEA IgG4 were significantly higher in sera from patients with fibrosis as detected by ultrasonography compared with other patients [28]. Similarly, Bonnard et al. [29] reported that higher levels of IgG4 and IgA against SEA antigens were found in patients with severe schistosomiasis. Moreover, the authors [28], [29] also showed no association between IgE responsiveness measured by direct ELISA against SEA antigens and disease severity. We demonstrated that IgG responsiveness to the *S. mansoni* antigens, SEA and SWAP, is also positively associated with severe forms of schistosomiasis, defined by clinical examination and by ultrasound measurement. Specifically, we identified an independent positive association between levels of anti-SWAP IgG and portal hypertension and fibrosis markers, such as thickness of the portal vein at its entrance into the porta hepatis and its bifurcation inside the liver and spleen size measured by ultrasound. Unsurprisingly, parasite-reactive IgG was also associated with disease because IgG4 recognizing SEA and SWAP antigens represent the most prevalent IgG isotype in *Schistosoma*-infected patients [29].

The association of IgG4 with severe forms of schistosomiasis has been justified by the increased susceptibility of the host to infection with the parasite; this finding indicates that an excess of IgG4 would block the protective effect of IgE, favoring parasite establishment and increasing egg deposition into host tissue and the progression to severe forms of the disease [26], [57], [58]. However, the positive association between IgG levels and disease in this study was independent of parasite burden, indicating that high infection intensity does not justify the more severe pathology found in individuals with strong IgG responses. Interestingly, Silveira et al. [28] described a positive association between anti-SEA IgG4 and fibrosis in individuals from endemic areas of schistosomiasis who were not excreting parasite eggs at the time of the examination, suggesting that IgG4 was associated with fibrosis rather than parasite burden.

Recent experimental work in mice [59] has demonstrated that blockage of IL-10 activity combined with PZQ treatment resulted in significant increases in the immune response and reductions in parasite burden during *S. mansoni* reinfection, associating IL-10 production with reinfection susceptibility. Interestingly, IL-10 is presumably needed to drive the differentiation of IgG4-switched B cells to IgG4-secreting plasma cells [60]. Moreover, Meiler et al. [61] demonstrated that regulatory T cells directly influence B cells. Regulatory T cell subsets lowered the frequency of IgE-secreting cells and simultaneously augmented the IgG4-secreting plasma cell frequency. Therefore, we propose that individuals with high levels of reactive IgG4 and low IgE are more susceptible to frequent reinfection that would favor more severe schistosomiasis, even in states of low parasite burden. This hypothesis also suggests a more complex role for IL-10 in *Schistosoma* pathology, with IL-10 acting as an anti-inflammatory factor during granuloma formation in the liver [20], [62], and as a modulator of the protective mechanism against re-infection [59].

Our data also showed, for the first time, an independent inverse correlation between anti-SEA and anti-SWAP IgE and the wall thickness of gallbladder, an important marker of fibrosis [8], [30], [34], suggesting a direct role for IgE in the modulation of granuloma formation. In an experimental model, a modulatory role for Ig/FcR signaling in *Schistosoma*-induced liver pathology was previously demonstrated by exacerbated hepatic granuloma formation and fibrosis detected in B cell-deficient mice, mice lacking the common FcRγ chain and mice deficient in FcεRI, the high-affinity receptor for IgE [24], [25], [64], [65]. In all the deficient mice mentioned, the authors reported severe schistosomiasis with similar patterns that were not related to parasite burden, differences in the T cell compartment or type-2 activation. These data suggest the possible participation of IgE/FcεRI and/or IgG-immune complexes/FcγRIII in triggering the production of anti-inflammatory mediators from FcR^+^ cells, such as FcR-bearing macrophages, DCs, B cells, basophils, mast cells and neutrophils. IC-FcγR activation could inhibit granuloma formation by generating the production of immunoinhibitory molecules, such as IL-10, prostaglandins and histamine, secreted by one or more of these types of cells [66]. More recently, experimental data [25] have indicated that during chronic *S. mansoni* infection, alternatively activated macrophages in the liver bind to immune complexes via FcRs and assume regulatory/anti-inflammatory roles. In the human population, a possible role for immune-complexes in granuloma formation/modulation was also suggested by experimental work that demonstrated that circulating immune complexes purified from the serum of patients with mild clinical forms of schistosomiasis downregulate the reactivity of peripheral blood mononuclear cells to Sepharose-conjugated parasite antigens, resulting in diminished granuloma reaction [67]. In contrast to immune complexes, a direct role for IgE in *S. mansoni* pathology was not reported for humans. Interestingly, histamine, which is normally associated with the induction of inflammation, has been shown in previous studies to downregulate schistosome-mediated granuloma formation [68]; therefore, this molecule is an important candidate for an FcR-induced pharmacological mediator of this process.

In summary, the current work demonstrated that parasite-reactive IgG levels are associated with signals of disease severity independently of parasite burden and that IgE concentration is inversely associated with *Schistosoma-*induced fibrosis. These data indicate an important role for antibodies in granuloma modulation, and consequently correlating with schistosomiasis severity.

## Supporting Information

Checklist S1 **STROBE Statement–Checklist of items included in reports of cross-sectional studies.**
(DOCX)
